# Wind-Mediated Spread of Low-Pathogenic Avian Influenza Virus into the Environment during Outbreaks at Commercial Poultry Farms

**DOI:** 10.1371/journal.pone.0125401

**Published:** 2015-05-06

**Authors:** Marcel Jonges, Jeroen van Leuken, Inge Wouters, Guus Koch, Adam Meijer, Marion Koopmans

**Affiliations:** 1 Centre for Infectious Disease Control, National Institute for Public Health and the Environment, Bilthoven, The Netherlands; 2 Department of Viroscience, Erasmus MC, Rotterdam, The Netherlands; 3 Institute for Risk Assessment Sciences, Faculty of Veterinary Sciences, Utrecht University, Utrecht, The Netherlands; 4 Central Veterinary Institute, Wageningen University & Research Center, Lelystad, The Netherlands; The University of Hong Kong, HONG KONG

## Abstract

Avian influenza virus-infected poultry can release a large amount of virus-contaminated droppings that serve as sources of infection for susceptible birds. Much research so far has focused on virus spread within flocks. However, as fecal material or manure is a major constituent of airborne poultry dust, virus-contaminated particulate matter from infected flocks may be dispersed into the environment. We collected samples of suspended particulate matter, or the inhalable dust fraction, inside, upwind and downwind of buildings holding poultry infected with low-pathogenic avian influenza virus, and tested them for the presence of endotoxins and influenza virus to characterize the potential impact of airborne influenza virus transmission during outbreaks at commercial poultry farms. Influenza viruses were detected by RT-PCR in filter-rinse fluids collected up to 60 meters downwind from the barns, but virus isolation did not yield any isolates. Viral loads in the air samples were low and beyond the limit of RT-PCR quantification except for one in-barn measurement showing a virus concentration of 8.48x10^4^ genome copies/m^3^. Air samples taken outside poultry barns had endotoxin concentrations of ~50 EU/m^3^ that declined with increasing distance from the barn. Atmospheric dispersion modeling of particulate matter, using location-specific meteorological data for the sampling days, demonstrated a positive correlation between endotoxin measurements and modeled particulate matter concentrations, with an R^2^ varying from 0.59 to 0.88. Our data suggest that areas at high risk for human or animal exposure to airborne influenza viruses can be modeled during an outbreak to allow directed interventions following targeted surveillance.

## Introduction

Avian influenza A viruses are highly heterogeneous, with varying pathogenicity across different species. They are classified into subtypes based on the surface glycoproteins haemagglutinin (HA) and neuraminidase (NA). Pathogenicity of the virus in chickens is related to the pathotype: low-pathogenic avian influenza (LPAI) viruses can contain any type of HA, while highly pathogenic avian influenza (HPAI) viruses invariably contain H5 or H7 [1]. Mortality is a prominent sign of HPAI-infected flocks, whereas LPAI-infected flocks show milder or even subclinical signs that can wax and wane, making LPAI more difficult to detect.

In wild birds, avian influenza virus is primarily transmitted through fecally contaminated surface water in shared aquatic habitats. In these habitats, the viruses can persist for extended periods, depending on water temperature and physico-chemical characteristics [2]. In domesticated birds, or poultry, HPAI viruses are typically found in both feces and respiratory secretions, while LPAI viruses are mainly shed through the enteric route [3]. Virus-contaminated droppings serve as source of infection for susceptible birds, and influenza viruses can remain infectious for many days in poultry litter [4, 5]. Dispersal of infectious material into the environment may occur through ventilation of virus-contaminated dust. In commercial poultry operations, concentrations of airborne dust are high and include a large component of fecal material along with food, dander (skin material), feather material, and micro-organisms [6]. Detection of influenza A virus in air measurements collected within farms suggest that particulate matter from infected poultry may play a role in avian influenza virus transmission to humans and birds, and other animals [7, 8].

One of the routes for pathogen transmission is through dispersal into outdoor air. Viruses may be dispersed as single particles or by using other particles (particulate matter) as a vehicle [9–11]. Recently, Ypma et al. estimated that wind direction could explain about 18% of the total transmission of avian influenza between farms during an outbreak of influenza A(H7N7) virus in 2003 [12]. However, wind direction alone does not quantify the amount of pathogen transmitted to a certain distance, as wind speed is another important factor [13]. Atmospheric dispersion models (ADMs) take these and other factors into account and have been applied to analyse the correlation between airborne pathogen transmission and the incidence of disease in the nearby surroundings for *Legionella pneumophila* [14], foot-and-mouth disease [15], *Coxiella burnetii* [16], and avian influenza virus [17]. Although the ADM data are suggestive of airborne pathogen dispersion, laboratory data have not yet confirmed that airborne avian influenza viruses are indeed detectable in the air downwind of a source.

Previously, we demonstrated that farm-to-farm spread of avian influenza viruses was associated with accumulated mutations that increase the public health risk of HPAI A(H7N7) viruses [18]. In addition, LPAI virus replication in poultry may trigger the emergence of an HPAI variant by alteration of the HA cleavage site, facilitating systemic infections. The consequent importance of early control of outbreaks became very clear with the 2013 emergence of avian influenza A(H7N9) viruses in China. Despite causing severe illness in humans, these viruses have the LPAI phenotype, making it hard to identify the avian sources and rendering humans as sentinels [19–21]. Gaining more insight into the transmission routes of avian influenza will help provide a more solid basis for current outbreak response strategies, and thereby could eventually reduce the public health risk associated with outbreaks.

In this study, we collected samples of suspended particulate matter, or inhalable dust fraction, inside, upwind and at several distances downwind of buildings holding poultry infected with LPAI. The samples were tested for the presence of influenza virus and for endotoxins, a marker for microbial exposure of poultry and livestock, since they have a high presence in commercial farms and can be quantified in the adjacent outdoor air [22]. We hypothesized that particulate matter may be used as a substitute for dispersion monitoring of avian influenza transmitted into the environment during outbreaks [10]. Consequently, airborne microbial exposure was determined by measuring endotoxin concentrations at different distances from farms and compared with an ADM to test the applicability of this model for the rapid characterization of a geographical region exposed during future outbreaks of avian influenza.

## Materials and Methods

### Farm description

At the following five LPAI-infected farms and one control farm, air samples were taken at multiple distances from the farms.

Farm 1 was a naturally ventilated organic chicken farm composed of an indoor-housed flock and a free-range flock. An LPAI A(H7N7) virus infection was detected by targeted investigation following signs of reduced food consumption, diarrhea, and limited growth of 8900 22-week old chickens. All chickens were culled on the day of confirmation of virus presence, in accordance with European guidelines for avian influenza virus subtypes H5 and H7 in commercial poultry (EU directive 92/40/EEC). Outdoor air sampling was initiated approximately six hours after the culling, at locations upwind and downwind of the farm.

Farm 2 was a mechanically ventilated turkey farm with 20,600 one-month old turkey chicks. It was tested for the presence of influenza virus because of negative health reports, showing infection with LPAI A(H9N2) virus. Nine days after sampling of the birds, air sampling was performed at locations upwind and downwind of the farm. No control measures were applied following outbreak confirmation, in accordance with EU guidelines.

Farm 3 was a bird-breeding farm that also housed various mammals and reptiles. Air was sampled upwind and downwind of 83 healthy-appearing wild swans that had been captured and were destined for export to a foreign zoo. The swans were quarantined following a positive screen for LPAI A(H5N2) virus performed as part of export guidelines. The air sampling was performed eleven days after A(H5N2) virus-positive cloaca swabs were collected. Twenty-four days after the initial A(H5N2) virus was detected, cloaca swabs indicated a continuing infection.

Farm 4 was a mechanically ventilated turkey farm housing three flocks: two with a total of 4000 21-week-old hens and one with 18,000 one-week-old chicks. In the hens, an LPAI A(H10N9) virus infection was detected following reports of reduced food consumption, respiratory signs including coughing, and malaise. Air sampling was performed at downwind locations nine days after the A(H10N9) virus-positive cloaca and trachea swabs were collected. No control measures were applied following outbreak confirmation.

Farm 5 was a mechanically ventilated mixed farm composed of two turkey flocks and a number of pigs. One turkey flock included 4000 20-week-old turkey cocks; the other included an unknown number of chicks. An LPAI A(H10N9) virus infection was detected following reports of increased mortality, nasal discharge, and respiratory signs in the 20-week-old turkeys. Air sampling inside and at downwind locations of the barn was performed three days after the A(H10N9) virus-positive cloaca and trachea swabs were collected. No control measures were applied following outbreak confirmation.

Farm 6, included as a control, was a naturally ventilated turkey farm that housed 16,500 one-month-old chicks. It was chosen because it was relatively isolated, with no commercial turkey farms (closest at ± 10 km) or chicken farms (closest at ± 4 km) in the immediate surroundings. In addition, the distance to nearest other livestock farms was >1 km. Air sampling was performed at downwind locations.

### Environmental samples

Environmental sampling was performed either on private land with permission of the owner or on public roads requiring no permissions. The experiment did not involve endangered or protected species.

Airborne inhalable dust samples were initially captured on a 37-mm diameter Teflon filter with a pore-size of 2.0 μm (SKC, PA, USA) using the GSP personal sampler (JS Holdings, Stevenage, UK) equipped with a conical inlet with an 8-mm diameter orifice at the front. The sampler meets the CEN/ISO/ACGIH criterion for inhalable dust when operated at 3.5 L per minute, which was achieved with a constant-flow pump (Gill air 5, Gillian, UK). Using a tripod, sampling was performed 1.5 m above ground for a six-hour period, resulting in a filtered-air volume of 1.3 m^3^. Multiple GSP samplers were used for simultaneous collection of inhalable dust samples at several distances from a farm. Immediately after sampling, the GSP sampling heads were wrapped in plastic before transport from field to laboratory, where they were stored at −20°C until further use.

In addition to the six-hour GSP air-sampling strategy, a short-term strategy using a portable air sampler was incorporated halfway into this study. The short-term air samples were obtained with an MD8-AirPort Air Sampler (Sartorius, Göttingen, Germany) equipped with cellulose nitrate filters having a pore size of 8 μm. This sampler was operated at 50 L per minute, with a sampling time of 20 minutes, resulting in a filtered-air volume of 1.0 m^3^. One MD8-AirPort sampler was used for consecutive collection of air samples at several distances from a farm. Immediately after sampling, each cellulose nitrate filter was transferred to a sterile Petri dish before transport from field to laboratory and storage at −20°C [23, 24].

### Influenza virus recovery

Our procedure for the detection of airborne influenza viruses was adopted from knowledge gained during a Q fever outbreak. We therefore evaluated whether the method for detecting *Coxiella burnetii* DNA in inhalable airborne dust collected on Teflon filters could be used for recovery of influenza virus by reverse transcriptase PCR (RT-PCR), using cell-culture grown influenza virus as a control [25]. We used three additional filter extraction procedures to determine which allowed the most sensitive RT-PCR detection of influenza viruses on filters.

To each filter we applied 20 individual 5-μL drops of heat-inactivated LPAI virus A/Mallard/NL/12/2000 (H7N3) in Dulbecco's modified Eagle medium (Gibco, NY, USA), corresponding with 1.3 x 10^5^ influenza genome copies [26]. The filters were air-dried and shaken for one hour in 4 mL pyrogen-free water with 0.05% Tween 20 (Calbiochem, CA, USA), with (method A) or without (method B) subsequent enzyme treatment intended to free bacterial DNA. Enzyme treatment consisted of adding 100 μL of 1 mg/mL lysostaphin (Sigma, MO, USA) and 20 μL of 20 mg/mL lysozyme (Sigma) followed by incubation for 35 minutes at 37°C, after which 400 μL of 20 mg/mL proteinase K (Roche diagnostics, Rotkreuz, Switzerland) was added and incubated for 10 minutes at 55°C. Enzymes were then heat-inactivated at 95°C for 10 minutes. This step was followed by DNA/RNA extraction using the NucliSens Magnetic Extraction Kit (bioMérieux, Marcy-l'Etoile, France) according to the manufacturer’s instructions [25].

Alternatively, spiked filters transferred to 2mL Eppendorf tubes containing 1 mL PBS and 1% Triton X-100 (BDH Chemicals, Poole, UK) were vortexed 3x10 seconds (method C), were mixed using a bench rocker for 30 minutes (method D) or sonicated for 30 minutes (method E), followed by RNA extraction as described below. For use as reference material, 100 μL influenza A/Mallard/NL/12/2000 (H7N3) virus was directly resuspended in 900 μL PBS and 1% Triton X-100. All procedures were performed *in triplicate*.

The presence of influenza virus was measured by a real-time influenza virus-specific RT-PCR as described below [26]. All obtained cycle-threshold (Ct) values were within the linear part (R^2^ = 0.9995) of the RT-PCR amplification. Influenza virus recovery was calculated by comparing the averaged Ct values per extraction procedure with the averaged Ct values of the reference material. The method with highest recovery was used in subsequent experiments.

### Environmental air sample processing

The Teflon filters (37mm diameter) collected from the GSP after air sampling were cut in half and transferred to 2 mL Eppendorf tubes prefilled with either 1 mL PBS containing 1% Triton X-100 or 1.5 mL infection medium consisting of Modified Eagle Medium with Hanks' BSS (BioWhittaker, Verviers, Belgium) supplemented with 10% PGR-albumin, penicillin, streptomycin, nystatin, L-glutamine, HEPES, and trypsin. The larger cellulose nitrate filters (80mm diameter) were likewise cut in half and transferred to 15-mL Greiner tubes prefilled with either 1.5 mL PBS containing 1% Triton X-100 for molecular testing, or 5 mL infection medium. Filters in infection medium were vortexed for 10 seconds followed by 0.22 μm filtration, and 300 μL (Teflon) or 2 mL (cellulose nitrate) of the flow-through was subsequently used for virus isolation.

### Detection of influenza virus

RNA was extracted from 600 μL of the recovered fluids from Triton X-100-treated Teflon and cellulose nitrate filters using the High Pure RNA isolation Kit (Roche), and influenza virus real- time RT-PCR was used to detect the matrix gene of the influenza virus [26, 27]. The influenza virus RT-PCR had a linear amplification range up to Ct value 31.15, corresponding with a limit of quantification of 1.1 x 10^4^ genome copies per ml or 3.0 x 10^2^ 50% egg infectious dose (EID50) per ml. The detection limit of the influenza virus RT-PCR was 320 genome copies per ml or 8.9 EID50 per ml.

### Influenza virus isolation

Filter-rinse fluids in infection medium were cultured on tertiary cynomolgus monkey kidney cells [28] and maintained in culture for a maximum of 2 weeks, or until cytopathic effect was observed. Presence of influenza virus in the culture supernatants was verified by RT-PCR as described above. The foregoing is standard procedure for human influenza virus isolation in our laboratory, which was proven effective for the isolation of avian influenza viruses during the influenza A(H7N7) virus outbreak in the Netherlands in 2003 [29]. Consequently, we hypothesized that it could be used to isolate avian influenza virus from filter fluids.

### Endotoxin measurement

In addition to influenza virus RNA, air samples obtained from farms 4, 5 and 6 were assayed for endotoxins, which can serve as a generic proxy for airborne poultry and livestock associated microbial exposure [22]. Endotoxin content of fluids from Triton X-100-rinsed filters was analyzed by the quantitative kinetic chromogenic Limulus amebocyte lysate (LAL) assay, described previously [30]. Inhibition or enhancement of the LAL-assay by application of 1% Triton X-100 was verified in dilution series, but was not observed when samples were diluted at least 1:50 in the assay. Consequently, fluids from Triton X-100-rinsed filters were tested in a dilution of 1:50 or higher. Results were expressed as endotoxin units (EU) per m^3^ (18EU = 1ng). The limit of detection was 1 and 2 EU per m^3^ of filtered air for the GSP and MD8-AirPort measurements, respectively.

### Detection of turkey cells

In addition to the quantification of endotoxins as a generic proxy for airborne microbial exposure, PCR detection of the *Meleagris gallopavo* (turkey) gene for mitochondria cytochrome oxidase 1 (CO1) was performed as a turkey farm-specific proxy for airborne exposure. Of fluids from Triton X-100-rinsed filters, 200 μL was used for automated total nucleic acid isolation on a MagNA Pure 96 extraction robot (Roche) with the MagNA Pure 96 DNA and Viral NA Small Volume Kit. Next, a real-time PCR assay was performed, targeting a 90-nucleotide fragment of the CO1 gene, using LightCycler 480 DNA SYBR Green I Master and the primers TurkeyCOI-F (5’-ACAACCATATTCTTATCATTAACC-3’) and TurkeyCOI-R (5’-GTTGCATTAAGTATAGGTGTTT-3’).

### Atmospheric dispersion model (ADM)

We compared the endotoxin measurements to relative spatial particulate matter concentrations calculated by the atmospheric dispersion model OPS-ST (Operational Priority Substances, Short Term) model, version 4. This ADM was developed by the Dutch National Institute for Public Health and the Environment (RIVM) for the dispersion modeling of chemical pollutants, i.e. particulate matter and ammonia/nitrogen oxides generated by traffic, industries, agriculture, and natural sources [31–33]. The OPS-ST model downloaded hourly-averaged meteorological data from the Royal Netherlands Meteorological Institute webserver, including wind speed, wind direction, solar radiation, temperature, precipitation amounts, and precipitation duration [33, 34]. We used coarse particulate matter as a proxy for endotoxin and assumed an environmental roughness length of 20 cm.

We defined the farms under investigation as point sources in the OPS-ST model, with arbitrary PM_10_ emission amounts per hour per source from 10:00AM to 16:00PM on sampling days. We calculated the concentrations at an above-ground height of 1.5 m on a grid of 2 x 2 km, with a grid-cell size of 10 m. Since we converted the modeled concentration levels relative to the concentration near the source, we were able to compare these modeled data to measured endotoxin concentrations by performing a linear regression analysis.

## Results

### Influenza virus recovery

Influenza virus recovery measured by RT-PCR was 10% when Teflon filters were processed by using method A, but recovery increased to 43% in the absence of enzyme treatment (method B). Rinsing of filters with PBS containing 1% Triton X-100 using the alternatives of vortexing (method C), a bench rocker (method D), or sonication (method E) resulted in influenza virus recovery of 60%, 26% and 57%, respectively. Based on these results, all filters were processed using method C prior to RNA extraction and influenza virus detection by RT-PCR.

### Influenza virus detection

As the applied influenza A virus RT-PCR demonstrated a limit of detection of approximately 6 genome copies per reaction, the processing of filter-rinse fluids combined with the available virus recovery data led to a theoretical limit of detection of approximately 300 and 500 influenza genome copies per m^3^ of filtered air for the GSP and MD8-AirPort measurements, respectively. Influenza A viruses could be detected by RT-PCR in outdoor-air samples obtained up to 60 meters downwind of commercial turkey farms 2, 4 and 5, which had ongoing LPAI infection (Table 1). However, the corresponding Ct values were high and beyond the linear RT-PCR amplification range (Ct value > 31.15), hampering virus quantification except for an indoor-air sample from farm 5 that had an influenza virus concentration of 8.48 x 10^4^ genome copies per m^3^. The air measurements initiated approximately six hours after the culling of farm 1 yielded no influenza virus. In addition, none were detected in the filter fluids from the air measurements obtained near the LPAI-positive swans at farm 3 or near control farm 6. Despite the fact that some of the air filters tested positive for influenza virus RNA, the GSP and MD8-AirPort filters yielded no virus isolates.

**Table 1:** Combined dataset depicting the farms, air-sample type, and location relative to the barn, and corresponding laboratory results including influenza virus RT-PCR detection, turkey COI RT-PCR detection, endotoxin quantification, and modeled relative particulate matter concentrations of all GSP and MD8-AirPort filters assayed.

Farm No.	species	virus subtype	air sample type	distance from barn	bearing from barn	type of measurement	influenza virus (Ct value)	turkey COI (Ct value)	Endotoxin EU/m3	OPS conc. (log)
1	chickens	H7N7	GSP: Teflon	42m	93°	downwind	neg	nd	nd	nd
			GSP: Teflon	53m	61°	downwind	neg	nd	nd	nd
			GSP: Teflon	150m	95°	downwind	neg	nd	nd	nd
			GSP: Teflon	200m	86°	downwind	neg	nd	nd	nd
			GSP: Teflon	190m	232°	upwind control	neg	nd	nd	nd
2	turkeys	H9N2	GSP: Teflon	40m	81°	downwind	36.59	nd	nd	nd
			GSP: Teflon	44m	129°	downwind	38.91	nd	nd	nd
			GSP: Teflon	58m	44°	downwind	37.76	nd	nd	nd
			GSP: Teflon	560m	91°	downwind	neg	nd	nd	nd
			GSP: Teflon	320m	143°	upwind control	neg	nd	nd	nd
3	swans	H5N2	GSP: Teflon	4m	30°	downwind	neg	nd	nd	nd
			GSP: Teflon	20m	77°	downwind	neg	nd	nd	nd
			GSP: Teflon	20m	348°	downwind	neg	nd	nd	nd
			GSP: Teflon	60m	70°	downwind	neg	nd	nd	nd
			GSP: Teflon	98m	54°	downwind	neg	nd	nd	nd
			GSP: Teflon	290m	177°	upwind control	neg	nd	nd	nd
4	turkeys	H10N9	GSP: Teflon	7m	56°	downwind	34.00	32.10	56.99	-0.42
			GSP: Teflon	8m	109°	downwind	35.81	33.83	26.14	-0.42
			GSP: Teflon	54m	59°	downwind	34.97	neg	8.60	-1.17
			GSP: Teflon	100m	53°	downwind	neg	neg	6.44	-1.56
5	turkeys	H10N9	MD8: Cellulose nitrate	0m		inside turkey stable	30.54	31.05	339.99	0.00
			GSP: Teflon	15m	104°	downwind	32.96	40.00	31.93	-0.76
			GSP: Teflon	28m	130°	downwind	36.3	neg	7.10	nd#
			MD8: Cellulose nitrate	44m	64°	downwind	35.94	neg	111.42	-1.01
			GSP: Teflon	47m	71°	downwind	34.31	40.00	48.31	-1.01
			MD8: Cellulose nitrate	54m	36°	downwind	36.38	neg	71.89	-1.18
			GSP: Teflon	59m	30°	downwind	34.56	neg	13.71	-1.37
			MD8: Cellulose nitrate	walkabout		downwind	neg	neg	96.72	nd
			MD8: Cellulose nitrate	0m		inside pig stable	neg	neg	98990.06	nd
			MD8: Cellulose nitrate	0m		inside turkey chick stable	neg	37.29	86.26	nd
6	turkeys	None	MD8: Cellulose nitrate	37m	15°	downwind	neg	neg	6.97	-2.21
			MD8: Cellulose nitrate	38m	24°	downwind	neg	neg	31.59	-1.09
			MD8: Cellulose nitrate	44m	42°	downwind	neg	neg	15.21	-1.00
			MD8: Cellulose nitrate	100m	47°	downwind	neg	neg	7.47	-1.89
			MD8: Cellulose nitrate	110m	59°	downwind; rain	neg	neg	2.60	-1.59
			MD8: Cellulose nitrate	110m	50°	downwind	neg	neg	7.07	-1.81
			MD8: Cellulose nitrate	160m	85°	downwind	neg	neg	5.00	-1.45
			MD8: Cellulose nitrate	190m	40°	downwind	neg	neg	*	-2.35
			MD8: Cellulose nitrate	200m	55°	downwind	neg	neg	*	-2.18
			MD8: Cellulose nitrate	410m	71°	downwind	neg	neg	3.82	-2.37

nd) not determined

*) Below detection limit

#) outside plume

### Endotoxin measurement

**Fig 1:**
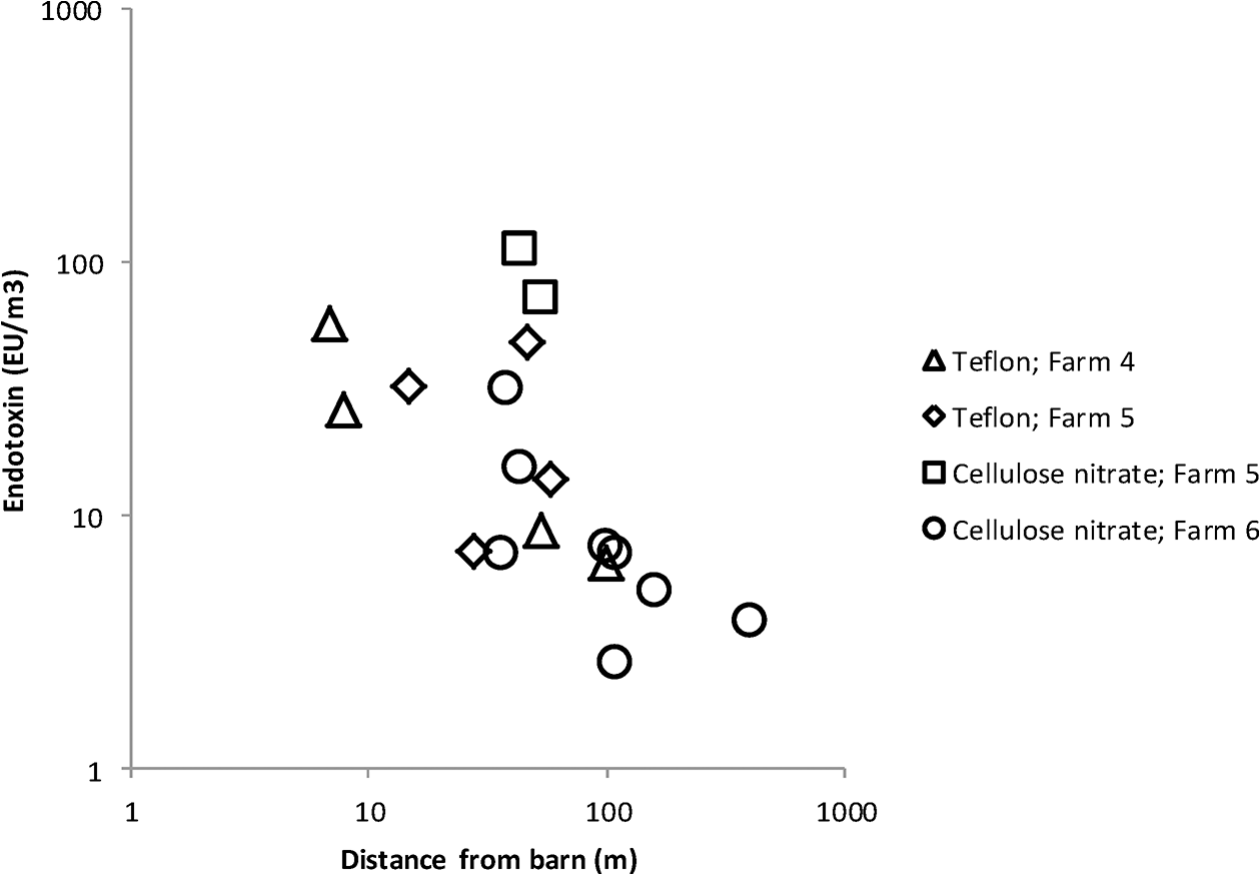
Endotoxin concentrations in air samples outside poultry barns are depicted in relation to the distance from the poultry barn, illustrating a reduction of airborne endotoxin with increasing distance from the source.

For farms 4, 5 and 6, fluids from Triton X-100-rinsed filters were likewise tested for the amount of air-suspended endotoxins (Table 1; Fig 1). As expected, endotoxin concentrations decreased as function of distance from the source, suggesting a reduction in microbial exposure with increasing distance. Air samples taken outside poultry barns had endotoxin concentrations of ~50 EU/m^3^ at distances up to 50 meters from the farm. At 100 meters and further from the farm, endotoxin concentrations decreased to <10 EU/m^3^. The highest endotoxin concentrations detected in outdoor-air samples corresponded with two MD8-AirPort measurements at poultry farm 5, probably influenced by emissions from a nearby upwind-located pig shed with an indoor endotoxin level of 99.000 EU/m^3^.

### Detection of turkey cells

The presence of air-suspended turkey cells was confirmed in samples obtained from farms 4 and 5 (Table 1). The three downwind dust samples collected closest to the farm tested positive for the turkey cells. However, at distances ≥ 10 m, turkey cell concentrations reached the limit of CO1 detection.

### Atmospheric dispersion modeling of particulate matter

**Fig 2:**
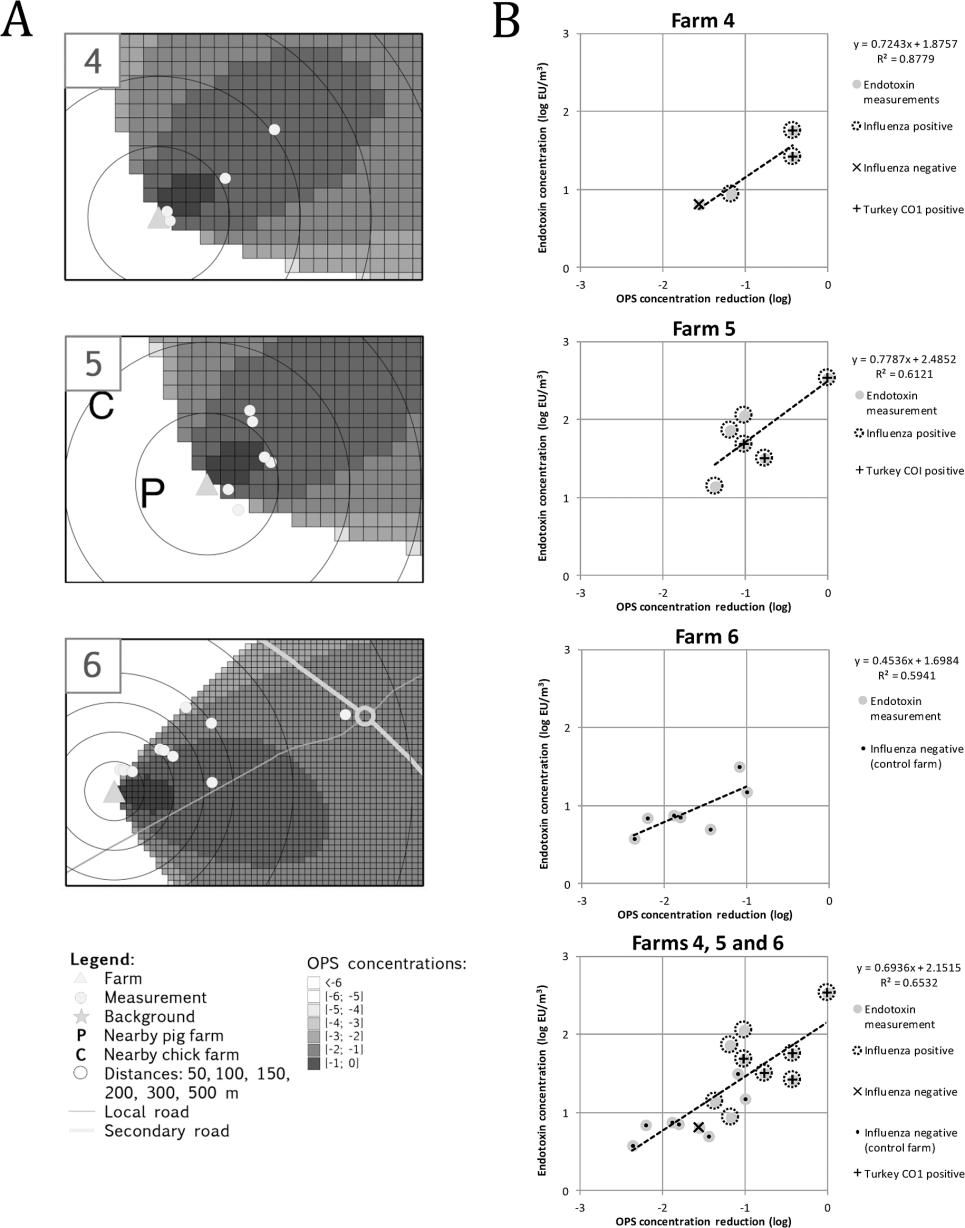
Dispersion of particulate matter around poultry farms, based on field measurements of endotoxin concentrations in air samples and OPS-ST particulate matter modeling. A) Maps illustrating the air sampling locations together with the atmospheric dispersion of particulate matter (relative to the source) that was modeled using meteorological data corresponding with the day and timeframe (10:00AM—16:00PM) of air sampling. B) Scatterplot of modeled dispersion and measured endotoxin concentration. Qualitative results of influenza virus RNA and turkey cell DNA detection are depicted as well.

Fig 2A shows the modeled relative concentrations of particulate matter at farms 4, 5 and 6. We next determined the modeled concentrations at the locations of air sampling (Table 1) and plotted them against the corresponding endotoxin concentration of each air filter location (Fig 2B). Air filters exposed to rain, located outside the dust plume, or showing an endotoxin concentration below the limit of detection were excluded. In general, endotoxin measurements and modeled relative concentrations of particulate matter showed a good correlation: linear regression analysis for farms 4 and 5 resulted in slopes of 0.72 (95% CI: 0.09 – 1.55) and 0.78 (95% CI: 0.08 – 1.64), respectively, and an R^2^ of 0.88 and 0.61, respectively. Analysis of farm 6 resulted in a slope of 0.45 (95% CI: 0.02 – 0.88) and an R^2^ of 0.59. Combining the data of farms 4, 5 and 6 resulted in an overall slope of 0.69 (95% CI: 0.42 – 0.97) and an R^2^ of 0.65.

## Discussion

We demonstrate the wind-mediated spread of influenza virus-contaminated poultry dust into the environment during influenza outbreaks in commercial poultry farms based on detection of the air-suspended virus downwind of farms. The observed influenza virus concentration of 8.5 x 10^4^ genome copies per m^3^ air inside turkey farm 5 is in agreement with previous reports of 6.9 x 10^4^ influenza virus particles per m^3^ detected in chicken houses and 3.7 ×10^4^ particles per m^3^ in a chicken pen, respectively [7, 8]. As the mechanical ventilation rates of commercial poultry housing range from a minimum of 0.5 m^3^/kg/hr up to 4.0 m^3^/kg/hr, the amount of air that is forced into the environment was at least 40.000 m^3^ per hour for farm 5, corresponding with the emission of over 3 x 10^9^ influenza virus genome copies per hour. A very large volume of virus particles could be shed into the environment during an outbreak, particularly with a source of prolonged emission like farm 4, where the infection started two weeks before the positive air samples were obtained. Such geographic dispersal of airborne virus may explain the detection of influenza virus RNA in, for example, dust swabs and samples of soil and mud puddles taken in areas surrounding farms positive for influenza A(H5N1) virus [35].

A crucial question when using this data for risk assessment is whether the viruses remain infectious during dust-mediated dispersal. We did not detect any infectious virus in our study, perhaps due to the sampling procedure. Mandal and Brandl (2011) have shown that dehydration stress caused by filtration reduces survival rates of bacteria [36]. Likewise for influenza viruses, filtration was found to reduce viability [37, 38]. Even when air is not passed through a filter, infectious influenza virus can decay with a 1–2 log reduction after drying at room temperature [39, 40]. Filtration and the subsequent processing of samples is therefore not optimal for the detection of live microorganisms, although Spekreijse et al. demonstrated that using a less dehydrating filter medium like gelatin allows detection of infectious influenza viruses in air-filter samples [41]. In our study, environmental conditions could also have led to influenza virus inactivation. Finally, virus recovery may have been reduced by 0.22 μm syringe filtration of the inoculum prior to cell culture inoculation, to minimize bacterial contamination. Our results are in agreement with the RT-PCR detection of swine influenza at locations downwind from swine farms using liquid cyclonic collectors, for which virus infectivity could not be confirmed, possibly due to low virus loads combined with physical disruption of viruses during air sampling [42].

In addition to virus detection by RT-PCR, the presence of turkey-specific CO1 gene was confirmed by PCR. Although this proxy for airborne microbial exposure is specific for turkey (farms), it appeared to be less sensitive than the generic endotoxin proxy. Nevertheless, the association between detectable virus and host nucleic acids illustrated the potential application of CO1 gene sequencing to assess sources of zoonotic agents in environmental samples.

Data from the six-hour measurements with GSP personal samplers confirmed the presence of influenza virus in the inhalable dust fraction of outdoor air near infected poultry farms. In addition to the approximately 10^6^ microbial cells present in 10 m^3^ air that humans inhale during the course of a day [36], our data suggests that humans living downwind of influenza virus-positive farms are possibly exposed to these virus particles. Unfortunately, our use of low-volume air samplers resulted in the detection of unquantifiable influenza virus RNA in the outdoor air. The use of high-volume air samples will presumably provide data that are more robust and allow outdoor air characterization at larger distances from an infected barn. In addition to the characterization of wind-borne influenza virus exposure, determining the infectivity of wind-borne influenza virus is challenging. Due to the loss of virus viability by air sampling methods, placement of naïve sentinel animals at a grid downwind of infected poultry farms is the method of choice to obtain relevant and conclusive data on the public health impact of wind-borne influenza virus spread. Such data can also be gained by sero-surveillance studies of humans and animals previously exposed to the virus.

As the average size of single influenza virus particles is 80 – 120 nm, our capture of viruses with 2.0 μm and 8.0 μm pore-sized filters suggests that these virus particles are indeed dispersed using other particles (particulate matter) as a vehicle [9–11, 43]. However, the mesh of fibers in aerosol filters (including Teflon and cellulose nitrate filters) enable efficient collection of much smaller particles than their pore-size would indicate [44]. Consequently, more research is needed to characterize the particle size distribution in relation to the detection of influenza virus RNA. Although the amount of influenza virus RNA in outdoor air near barns was unquantifiable, airborne poultry and livestock associated microbial exposure was determined by measuring concentrations of endotoxin. Endotoxin concentrations measured at different distances from farms were compared with modeled concentrations of particulate matter. Despite the many variables that potentially influenced the relationship between emission of particles and concentration, a good correlation between field measurements and modeled particulate concentrations was observed (Fig 2B). Although the 95% confidence intervals of the slopes corresponding with individual farms were large, combining all farms reduced the confidence interval, possibly by averaging out errors.

Our results suggest that an ADM like the OPS-ST model could be used to model the dispersion of outdoor airborne pathogens prospectively. However, the quantitative model outcomes should be regarded as indicative, given the number of unknown uncertainty factors. For example, the model predicts dispersion of PM_10_ while it is yet unknown what proportion of influenza viruses are associated with the PM_10_ fraction of airborne particulate matter. Nor is it known how the pathogens are distributed over the different size fractions within PM_10_. Although an association was found between modeled and measured concentrations, the slopes in Fig 2B did deviate from 1, suggesting that dispersion is slightly different for endotoxins than for modeled dust. The difference should be clarified in future studies. Moreover, it should be noted that virus inactivation (e.g., as a result of UV-radiation or dehydration) is not included in the model. Since our measurements were performed at relatively short distances from the infected farms, absence of an inactivation rate will not lead to high biases. At larger distances, however, the inactivation rate will be more important and must be included when this type of information becomes available.

In accordance with EU directive 92/40/EEC, controlling outbreaks of HPAI viruses relies on movement restrictions for farms within a radius of at least 10 km of an infected farm, along with culling of infected poultry. Depending on the outbreak severity, additional control measures can be taken including preventive ring-culling of farms within a (1–5 km) radius of an infected farm and extended (nationwide) standstill for the transport of live poultry. Despite such measures, avian influenza virus outbreaks in areas with high concentrations of poultry have been difficult to control, resulting in large-scale culling in the Netherlands, Canada and Mexico [45–47]. More directed interventions can potentially limit the duration of the outbreak and the number of culled farms [48]. Gaining insight on farm-specific virus spread allows directed interventions following targeted surveillance.

In this study, we demonstrated the presence of airborne influenza virus RNA downwind from buildings holding LPAI-infected birds, and observed correlation between field data on airborne poultry and livestock associated microbial exposure and the OPS-ST model. These findings suggest that geographical estimates of areas at high risk for human and animal exposure to airborne influenza virus can be modeled during an outbreak, although additional field measurements are needed to validate this proposition. In addition, the outdoor detection of influenza virus-contaminated airborne dust during outbreaks in poultry suggests that practical measures can assist in the control of future influenza outbreaks.

In general, exposure to airborne influenza virus on commercial poultry farms could be reduced both by minimizing the initial generation of airborne particles and implementing methods for abatement of particles once generated [6, 49]. As an example, emergency mass culling of poultry using a foam blanket over the birds instead of labor-intensive whole-house gassing followed by ventilation reduces both exposure of cullers and dispersion of contaminated dust into the environment, contributing to the control of influenza outbreaks [50, 51].
